# Influence of dietary supplementation with new *Lactobacillus* strains on hematology, serum biochemistry, nutritional status, digestibility, enzyme activities, and immunity in dogs

**DOI:** 10.14202/vetworld.2023.834-843

**Published:** 2023-04-21

**Authors:** Kamonporn Panja, Sathita Areerat, Pipatpong Chundang, Pornsucha Palaseweenun, Nattaphong Akrimajirachoote, Jaruwan Sitdhipol, Punnathorn Thaveethaptaikul, Pennapa Chonpathompikunlert, Kanidta Niwasabutra, Pongsathon Phapugrangkul, Attawit Kovitvadhi

**Affiliations:** 1 Graduate Student in Animal Health and Biomedical Science Program, Faculty of Veterinary Medicine, Kasetsart University, Bangkok 10900, Thailand; 2 Faculty of Veterinary Medicine, Rajamangala University of Technology Tawan-ok, Bangpra, Chonburi 20110, Thailand; 3 Department of Physiology, Faculty of Veterinary Medicine, Kasetsart University, Bangkok 10900, Thailand; 4 Biodiversity Research Center, Thailand Institute of Scientific and Technological Research, Pathumthani 12120, Thailand; 5 Expert Center of Innovative Health Food, Thailand Institute of Scientific and Technological Research, Pathumthani 12120, Thailand

**Keywords:** digestibility, *Lactobacillus*, nutritional status, probiotics

## Abstract

**Background and Aim:**

The use of antibiotics is associated with many side effects, with the development of bacterial resistance being particularly important. It has been found that dogs and their owners host similar resistant bacteria. This contributes to increased concurrent bacterial resistance and a possible trend of increased bacterial resistance in humans. Thus, using probiotics in dogs is an alternative option for preventing and reducing the transmission of bacterial resistance from dogs to humans. Probiotics are characterized by their potential to endure low pH levels and high concentrations of bile acids in the gastrointestinal tract. Lactobacilli are more acid-tolerant and resistant to bile acid, so they are ideal probiotics to be added to the canine diet. According to the previous studies, the benefits of *Lactobacillus* are a stable nutritional status and greater digestibility, along with improved fecal scores and reduced ammonia in dogs. However, no studies have been conducted with *Lactobacillus plantarum* CM20-8 (TISTR 2676), *Lactobacillus acidophilus* Im10 (TISTR 2734), *Lactobacillus rhamnosus* L12-2 (TISTR 2716), *Lactobacillus paracasei* KT-5 (TISTR 2688), and *Lactobacillus fermentum* CM14-8 (TISTR 2720), or their use in combination. Hence, the aim of this study was to examine the possible effects of the aforementioned *Lactobacillus* on hematological indices, nutritional status, digestibility, enzyme activities, and immunity in dogs. From the results, a new and safe strain of *Lactobacillus* may emerge for use as a probiotic in the future.

**Materials and Methods:**

In this study, 35 dogs were allocated equally into seven groups: Group 1 received a basal diet (control), while Groups 2–7 received the same diet further supplemented with *L. plantarum* CM20-8 (TISTR 2676), *L. acidophilus* Im10 (TISTR 2734), *L. rhamnosus* L12-2 (TISTR 2716), *L. paracasei* KT-5 (TISTR 2688), *L. fermentum* CM14-8 (TISTR 2720), or a mixture of probiotics (*L. plantarum*, *L. acidophilus*, *L. rhamnosus*, *L. paracasei*, and *L. fermentum*), respectively. All probiotics were administered at a dose of 10^9^ colony-forming unit/dog for 28 days. Nutritional status, hematology, serum biochemistry, digestibility, enzyme activities, and immunity parameters were assessed.

**Results:**

There were no differences among the groups in body weight, feed intake, body condition score, fecal score, and fecal dry matter on the different sampling days. The hematology and serum biochemical analyses showed a difference only in creatinine activity (p < 0.001), with higher values in group *L. fermentum* CM14-8 (TISTR 2720) and lower values in group *L. paracasei* KT-5 (TISTR 2688) than in controls. However, all measurements were within the normal laboratory reference ranges. Fecal characteristics (fecal ammonia and fecal pH), fecal digestive enzyme activities, serum immunoglobulin (IgG), and fecal IgA did not differ significantly among the groups (p > 0.05).

**Conclusion:**

*Lactobacillus plantarum* CM20-8 (TISTR 2676), *L. acidophilus* Im10 (TISTR 2734), *L. rhamnosus* L12-2 (TISTR 2716), *L. paracasei* KT-5 (TISTR 2688), and *L. fermentum* CM14-8 (TISTR 2720), along with their mixture are safe and non-pathogenic additives for use as new probiotic strains of *Lactobacillus* in dogs. Although the new *Lactobacillus* strains had no effect on hematology, serum biochemistry, nutritional status, digestive enzyme activities, immunity, body weight, feed intake, or body condition scores in dogs, further studies should investigate the intestinal microbiota and the development of clinical treatments.

## Introduction

Antibiotic use has many side effects, such as adverse changes in the intestinal microbiota [1], the presence of drug residues in the diet, immunopathological effects, and carcinogenicity [2]. In particular, the development of resistant bacteria causes drug-resistance problems, making infection challenging to control [3]. Studies have shown that dogs and their owners host similar resistant bacteria. This results in increased concurrent bacterial resistance and might lead to the expansion of bacterial resistance in humans. Against this background, probiotics are an alternative option to prevent and cure infections [4] by replacing antibiotic use in dogs. Probiotics are defined as live microorganisms used as supplements in the diet. They confer advantages to the host by controlling the intestinal microbiota and improving health [5, 6]. Moreover, they improve mucosal health through several mechanisms, for example, the replacement of intestinal pathogens [7], the creation of antimicrobial substances [8], the activation of immune responses [9], and the enhancement of various metabolites [10]. In addition, they produce lactic acid and synthesize enzymes, vitamins, and short-chain fatty acids [11–13]. These effects tend to lower lactose intolerance, modulate the immune system, and reduce the production of putrefactive compounds, such as ammonia, which affects fecal odor and colon carcinogenesis [5].

Among probiotic strains used in humans and animals, those commonly applied belong to the lactic acid bacteria (LAB) [14]. The majority of species that are found in the mammalian gastrointestinal tract (especially proximal small intestine) and form powerful attachments to the epithelium belong to the genus *Lactobacillus* [15]. *Lactobacillus* spp. are Gram-positive bacteria that are used in a wide range of marketed probiotics as well as industrial food and bioprocessing technology [16]. *In vitro* studies with *Lactobacillus* strains found that they suppressed the growth of pathogenic bacteria, such as *Escherichia coli* and *Clostridium difficile*, which have multi-drug-resistant properties encoded in their genomes [17]. Drug-resistance is induced via the occurrence of biofilm. Lactobacilli exert anti-biofilm-forming activities against *Staphylococcus aureus*, *Pseudomonas aeruginosa*, and *Enterococcus faecalis* [18]*. Lactobacillus rhamnosus* and *Lactobacillus casei* possess excellent antimicrobial activity by synthesizing organic acids and diminishing pH. These antimicrobial properties are important for slowing down the growth, proliferation, and colonization of pathogenic bacteria [19, 20]. *Lactobacillus* inhibits the adhesion of *E. coli* to epithelial cells and reduces their expression of the proinflammatory cytokine IL-8 on activation by *E. coli* [21]. Thus, this strain reduces drug resistance and inhibits pathogenic bacterial growth or attachment to epithelial cells. In a previous study, *Lactobacillus acidophilus* D2/CSL was administered to healthy dogs for 35 days at a concentration of 5 × 10^9^ colony-forming unit (CFU)/g. It improved fecal moisture, fecal score, and fecal hardness and also controlled weight [22]. *Lactobacillus plantarum* strain DSM13241 (>10^9^ CFU/g) improved red blood cell (RBC) count, hematocrit, and immunoglobulin (Ig)G in a 4-week trial [23]. *Lactobacillus plantarum* K10 exerted anti-obesity effects in mice when administered for a duration of 12 weeks [24]. Moreover, *L. acidophilus* (3.0 × 10^8^ CFU/mL) administration for 28 days reduced fecal ammonia without changing serum total cholesterol, white blood cell (WBC) count, red blood cell (RBC) count, or the blood lymphocyte percentage in healthy beagles [25] and raised digestibility in weaning pigs [26]. *Lactobacillus fermentum* AD1 (10^9^ CFU/g diet) can lead to the colonization of the canine gastrointestinal tract by *Lactobacillus*, leading to improvement of the fecal count of lactobacilli and an elevated serum level of total protein in healthy dogs [27]. *Lactobacillus plantarum* strain RW1 was also reported to reduce the release of proinflammatory cytokines from *Salmonella* spp. [28]. *Lactobacillus rhamnosus* MP01 and MP02 increased fecal *Lactobacillus* counts in dogs and provided protection against gastrointestinal infection [29]. *Lactobacillus johnsonii* CPN23 at a dose of 2–3 × 10^8^ CFU/day improved fiber digestibility and decreased ammonia production in adult female Labrador dogs in a 9-week experiment [30]. Another study of *L. johnsonii* CPN23 dietary supplementation (10^8^ CFU/mL; 0.1 mL/kg body weight) found that it reduced plasma glucose and cholesterol levels but increased total protein and the high-density lipoproteins/Low-density lipoprotein ratio in dogs [31]. Meanwhile, *Lactobacillus murinus* LbP2 (5 × 10^9^ CFU/day; 2 weeks) was reported to upregulate fecal IgA in healthy dogs. Many mixtures of probiotics have been used with powerful effects. A mixture of probiotics (*L. johnsonii* NCC2767, *L. acidophilus* NCC2628, and *L. acidophilus* NCC2766) applied at a concentration of 10^10^ CFU/strain/day in dogs decreased the expression of interleukin-10 (anti-inflammatory cytokine) and increased that of interferon-gamma (proinflammatory cytokine) [32]. Moreover, it was reported that a probiotic mixture (*Bifidobacterium animalis*, *L. plantarum*, and *L. casei*, used at a concentration of 2 × 10^9^ CFU/g for a duration of 60 days) increased IgG and secretory IgA in healthy dogs [33]. This may affect direct immunity (IgA) and indirect immunity (IgG). In dogs with food-responsive diarrhea, *L. acidophilus* (NCC2628, NCC2766) mixed with *L. johnsonii* (NCC2767) at a dose of 10^10^ CFU/g for 4 weeks decreased the *Enterobacteriaceae* count but increased the number of *Lactobacillus* spp. Furthermore, it improved the fecal score and Canine Inflammatory Bowel Disease Activity Index [32]. Elsewhere, milk fermentation product of *L. fermentum* VET 9A, *L. plantarum* VET 14A, and *L. rhamnosus* VET 16A was tested in acute diarrhea at 2 × 10^9^ CFU/mL. They improved the fecal score and feed intake in dogs [34]. Furthermore, mixed probiotics of *Bifidobacterium* and *Lactobacillus* at a dose of 3 × 10^10^ CFU/capsule following the manufacturer’s recommendations were used in canine acute diarrhea for 10 days and normalized the stool consistency [35]. Inflammatory bowel disease in dogs was also treated with a mixed strain product, which contained *Lactobacillus* (*L. acidophilus, Lactobacillus delbrueckii, L. plantarum*, and *L. casei*), *Bifidobacterium*, and *Streptococcus salivarius*, at a dose of 112–225 × 10^9^ CFU/10 kg/day for 60 days. The study found improvements in clinical and histological scores and an increase in T-cell regulatory markers (FoxP3 and TGF-β) [36]. Enzymes such as proteases, amylases, and cellulases are produced by LAB [37]. *Lactobacillus* is a group of gram-positive bacteria belonging to LAB [38]. *Lactobacillus plantarum* added to the diet enhanced dietary digestibility (protein, starch, and fat) and improved nutritional performance in common carp [39]. For these reasons, *Lactobacillus* may increase enzyme activity (amylase, protease, and cellulase) and is proposed to augment digestibility. Therefore, *Lactobacillus* spp. acts as an immunomodulator, improves fecal score and fecal moisture, reduces pH and ammonia, and increases digestibility. The optimal characteristics of probiotics are their potential to endure low pH levels in the stomach and high concentrations of bile acids in the intestinal tract [40, 41]. *Lactobacillus* spp. are more acid-tolerant and resistant to bile acid [42], making them appropriate probiotics for supplementation of the canine diet.

Interestingly, *L. plantarum* CM20-8 (TISTR 2676), *L. acidophilus Im10* (TISTR 2734), *L. rhamnosus* L12-2 (TISTR 2716), *Lactobacillus paracasei KT-5* (TISTR 2688), and *L. fermentum* CM14-8 (TISTR 2720) are new local probiotic strains that have never been studied in dogs for their efficiency and safety. Thus, our study aimed to examine the effects of supplementation with these probiotics on hematology, serum biochemistry, digestibility, enzyme activities, and immunity in dogs.

## Materials and Methods

### Ethical approval

The ethics committee of Kasetsart University, Bangkok, Thailand (ACKU64-VET-046), approved all procedures used in this study.

### Study period and location

The study was conducted from 13 July 2021 to 9 August 2021. The study was conducted at Canine experimental unit (Faculty of Veterinary Medicine, Kasetsart University, Nakhon Nayok, Thailand).

### Probiotics

*Lactobacillus plantarum* CM20-8 (TISTR 2676), *L. acidophilus* Im10 (TISTR 2734), *L. rhamnosus* L12-2 (TISTR 2716), L. paracasei KT-5 (TISTR 2688), and *L. fermentum* CM14-8 (TISTR 2720) were obtained from the Biodiversity Research Centre, Thailand Institute of Scientific and Technological Research, Pathumthani, Thailand. These probiotics are non-pathogenic in humans and animals. They tolerate gastric juice at pH 2.5 and bile acid at pH 8 for 180 *min* in the gastrointestinal tract. Furthermore, they inhibit *S. aureus, Salmonella* Enteritidis, Salmonella Typhimurium, and *E. coli*. They had already passed the *in vitro* test for antioxidant properties and immunomodulation by activating TGFb-α and IL-2 and inhibiting TNF-α and IL-6. The probiotics were cultured in De Man-Rogosa-Sharpe agar under facultative anaerobic conditions at 37°C. All probiotics were lyophilized to a powder. Then, they were mixed with maltodextrin to reach the final desired concentration and stored in vacuum bags at 4°C before use. All probiotic strains showed an adhesion level in the Caco-2 cell line of more than 70%. These are important characteristics of candidate probiotics [43]. Their results from antibiotic susceptibility tests showed resistance to aminoglycosides, quinolones, and vancomycin groups. From this pattern, these strains cannot pass on resistance to other microorganisms [44, 45]. However, these probiotics were susceptible to amoxicillin, ampicillin, erythromycin, penicillin, chloramphenicol, clindamycin, tetracycline, and imipenem. Therefore, these isolated probiotics were safe to be used in feed because they do not have any genes conferring resistance to these antibiotic groups [46].

### Animals

Thirty-five healthy, adult, mixed-breed dogs (17 males and 18 females), 2–4 years old, with an average body weight of 17.7 ± 0.76 kg and body condition score of 3.47 ± 0.28 (nine-point body condition score) [47], were randomly selected from an experimental farm. They had previously been vaccinated and dewormed and had passed a physical examination by a veterinarian. The dogs had not received antibiotics or any other medications for at least 3 months before and during the experiment. The animals were housed in 2.0 × 2.0 × 3.0 m individual cages located in the canine experimental unit (Nakhon Nayok, Thailand).

### Experimental design, diet, and feeding

The 35 dogs were randomly and equally allocated to seven experimental groups as follows: Group 1, fed a basal diet with top dressing by only maltodextrin as a placebo (Control); Group 2, fed a basal diet with top dressing by *L. plantarum* CM20-8 (TISTR 2676); Group 3, fed a basal diet with top dressing by *L. acidophilus* Im10 (TISTR 2734); Group 4, fed a basal diet with top dressing by *L. rhamnosus* L12-2 (TISTR 2716); Group 5, fed a diet with top dressing by *L. paracasei* KT-5 (TISTR 2688); Group 6, fed a basal diet with top dressing by *L. fermentum* CM14-8 (TISTR 2720); and Group 7, fed a basal diet with top dressing by a mixture of the abovementioned probiotics with an equal amount of each. The basal diet consisted of commercially extruded pellets (Ole Dog Beef flavor^®^; Greatest Pet Care Co., Ltd., Bangkok, Thailand) containing crude protein, crude fat, crude fiber, and crude ash at 19.56%, 10.83%, 6.69%, and 6.28% fresh matter, respectively. The daily energy requirement was calculated as 1.6 × 70 × body weight^0.75^ [48]. Water was provided *ad libitum*. Food and supplements were offered once daily at 15:00. The top dressing of probiotics (or placebo) was provided to dogs once a day and contained a probiotic dose of 1 × 10^9^ CFU. For digestibility traits, the adaptation phase was from days 0 to 22, and the collection phase was 5 days after the adaptation phase. The experiment was conducted for 28 days.

### Sample collection and analysis

Body weight and feed intake measurements, determination of nine-point body condition score [47], physical examination, and blood collection were performed on days 0, 14, and 28. Blood was collected from the cephalic vein into an ethylenediaminetetraacetic acid tube for hematological analysis, whereas another aliquot of blood was kept in a serum tube for the evaluation of blood chemistry blood urea nitrogen; creatinine; alanine aminotransferase; total protein; albumin; and IgG. Hematology and blood chemistry were analyzed at Veterinary Diagnostic Laboratory, Kasetsart University Veterinary Teaching Hospital, Faculty of Veterinary Medicine, Kasetsart University, Bangkok, Thailand. The serum concentration of IgG was evaluated using the Canine IgG ELISA Kit (Cat# ab157701; Abcam, Cambridge, MA, USA). Optical density (OD) was measured at 450 nm with a microplate reader and the IgG concentration was calculated in accordance with the manufacturer’s protocols.

Fecal samples were collected on days 0, 14, and 28 for analysis of the fecal score [49, 50] and dry matter, whereas fecal pH, ammonia, and digestive enzyme activities (amylase, protease, and cellulase) were evaluated on the same days. For the evaluation of digestive enzyme activities, 1 g of fecal sample was diluted in 10 mL of ice-cold phosphate-buffered saline (PBS, pH 7.0) and homogenized using a hand-held glass homogenizer. The homogenate was centrifuged at 18,000× *g* for 20 min at 4°C. The supernatants were divided into microcentrifuge tubes and stored at −20°C for analysis [51]. The supernatant was used for three enzyme assays: amylase activity (EC 3.2.1.1, substrate starch; Univar, Thermo Fisher, USA), cellulase activity [52, 53] (EC 3.2.1.4, carboxymethyl cellulose substrate; Univar), and protease activity using casein as the substrate [54]. The reaction products of amylase and cellulase were stained with 1% dinitrosalicylic acid and measured in a spectrophotometer at 540 nm with maltose and glucose as standards. Another product of protease activity was stained with 0.5 mM Folin-Ciolcalteu reagent. The absorbance of the mixture was determined at 610 nm with L-tyrosine as a standard.

Dry matter and the apparent nutrient digestibility of organic matter, crude protein, and ether extract were evaluated during days 23–28 of the experimental period. Feed intake and fecal output were also measured during this period. In addition, dry matter, organic matter, crude protein, and ether extract were analyzed in food and feces by following Association of Official Analytical Chemists protocols [55]. The results were transformed to calculate the dry matter and apparent nutrient digestibility.

Feces were collected on days 0 and 14 for the analysis of IgA. One g of feces was homogenized with 10 mL of extraction buffer (0.5% Tween; Sigma-Aldrich, Poole, UK; and 0.05% sodium azide in 0.01 M PBS, pH 7.4) in a vortex mixer. The sample suspensions were centrifuged at 1500 g for 15 min at 5°C. The supernatant (2 mL) was transferred to a clean test tube containing 20 μL of protease inhibitor cocktail (Sigma-Aldrich, St. Louis, MO, USA) [56, 57]. All samples were centrifuged and mixed at 10,000× *g* for 10 min. Then, the sample supernatants were transferred to sterile test tubes and stored at −20°C until IgA concentrations were measured. The concentration of IgA was evaluated using the Canine IgA ELISA Kit (Cat# ab157699; Abcam). Optical density was measured at 450 nm with a microplate reader and the IgA concentration was calculated in accordance with the manufacturer’s instructions.

### Statistical analysis

Ordinal data, body condition scores, and fecal scores were evaluated by the Kruskal–Wallis test with Dunnett’s test as post hoc analysis. A factorial experiment with a completely randomized design was used in this study. The hematology, blood chemistry, fecal pH, fecal ammonia, digestive enzyme activities, serum IgG, and fecal IgA were evaluated among the studied groups by two-way mixed model analysis of variance (ANOVA) with Duncan’s multiple range test as a post hoc analysis. The date of collection served as a random factor, whereas the treatment group was considered as a fixed factor.

One-way ANOVA with Duncan’s multiple range test as a post hoc analysis, in which the fixed factor was the studied groups, was used to identify differences in body weight, feed intake, fecal moisture, and apparent digestibility. The data were evaluated by the Shapiro–Wilk test and Levene’s test to confirm the normal distribution and homogeneity of variances, respectively. p < 0.05 indicated statistical significance. All statistical analyses were performed using R-statistic with the Rcmdr package in RStudio Desktop 2021.09.2+382 (R-Statistic, Vienna, Austria).

## Results

### Body weight, feed intake, body condition score, fecal score, and fecal moisture

No clinical signs were presented during the experiments based on physical examinations on days 0, 14, and 28. There were no differences among the groups in body weight, feed intake, body condition score, fecal score, and fecal moisture over the entire experimental period (Table-1).

**Table-1:** Effects of probiotic addition in dog food on the body weight, feed intake, nine-scale body condition score, fecal score, and fecal moisture.

Parameters	Groups^1^							SEM^2^	p-value

	1	2	3	4	5	6	7		
Body weight (kg)									
Day 0	16.2	16.9	16.1	19.4	16.1	18.6	20.8	0.756	0.52
Day 14	15.3	16.7	16.6	18.5	15.3	18.0	19.8	0.721	0.62
Day 28	15.2	16.4	17.2	19.3	15.3	17.5	20.4	0.765	0.51
Feed intake (g/d)									
Day 0	312	318	304	351	312	325	378	11.42	0.63
Day 14	292	308	294	339	294	307	340	9.817	0.74
Day 28	266	305	293	323	270	296	317	8.964	0.57
Nine scale body condition score									
Day 0	4.20	3.50	4.20	4.40	3.90	4.00	5.00	0.198	0.20
Day 14	4.30	4.00	4.40	4.50	4.00	4.40	5.10	0.198	0.65
Day 28	4.50	4.40	4.60	4.50	4.20	4.40	5.00	0.196	0.98
Fecal score									
Day 0	2.40	3.00	2.80	3.40	2.00	3.80	4.20	0.282	0.42
Day 14	2.20	2.75	2.50	3.20	2.40	2.80	3.40	0.151	0.18
Day 28	2.20	2.80	2.40	2.80	2.40	2.40	3.00	0.094	0.20
Fecal moisture (%)									
Day 0	62.2	67.0	64.4	66.0	67.5	66.6	68.2	0.778	0.42
Day 14	70.7	69.1	70.2	74.3	70.7	71.1	73.3	0.555	0.15
Day 28	70.8	66.8	67.6	70.0	68.7	66.8	72.9	0.647	0.08

1 Group 1=Control group (none of probiotic supplementation), Group 2=*Lactobacillus plantarum* CM20-8 (TISTR 2676), Group 3=*Lactobacillus acidophilus* Im10 (TISTR 2734), Group 4=*Lactobacillus rhamnosus* L12-2 (TISTR 2716), Group 5=*Lactobacillus paracasei* KT-5 (TISTR 2688), Group 6=*Lactobacillus fermentum* CM14-8 (TISTR 2720) and Group 7=Mixed of probiotics.

2 Standard error of the mean

### Hematology and serum biochemistry

The hematology and serum biochemical analyses for each group are summarized in Table-2. A difference in creatinine was found between the *L. paracasei* KT-5 (TISTR 2688) and *L. fermentum* CM14-8 (TISTR 2720) groups (p < 0.001). The creatinine activity of *L. paracasei* KT-5 (TISTR 2688) was lower than that of the control, whereas that of *L*. *fermentum* CM14-8 (TISTR 2720) was greater than that of the control. However, all groups were within the normal laboratory range for healthy dogs. The other parameters did not show any significant differences among the groups (p > 0.05).

**Table-2:** Effects of probiotic addition in dog food on the hematological and serum biochemical parameters.

Parameters^1^	Groups^2,3^							SEM	p-value	Reference range^4^

	1	2	3	4	5	6	7			
Hemoglobin (g/dL)	16.3	15.5	16.4	14.8	14.8	16.2	16.5	0.213	0.08	13.1–20.5
Hematocrit (%)	48.6	46.0	49.7	45.0	44.9	48.3	50.1	0.607	0.06	37.3–61.7
Red blood cell (10^6^/uL)	7.72	9.49	7.73	11.1	9.49	7.79	7.96	0.715	0.82	5.65–8.87
MCV (fL)	62.4	62.0	64.4	63.1	63.9	62.0	62.9	0.286	0.06	61.6–73.5
MCHC (g/dL)	33.7	55.4	33.2	33.0	32.9	33.5	33.0	3.163	0.43	32.0–37.9
MCH (pg)	21.0	20.9	21.4	20.8	21.0	20.8	20.7	0.101	0.17	21.2–25.9
White blood cell (10^3^/uL)	14.9	11.5	12.7	13.9	13.1	12.7	13.0	0.299	0.30	5.05–16.8
Neutrophils (10^3^/uL)	9.46	7.14	8.35	8.95	7.68	8.78	8.87	0.228	0.08	2.95–11.6
Lymphocytes (10^3^/uL)	3.56	2.75	2.73	2.61	2.99	2.23	2.39	0.121	0.15	1.05–5.10
Monocytes (10^3^/uL)	0.65	0.53	0.53	0.60	0.68	0.51	0.54	0.024	0.06	0.16–1.12
Eosinophils (10^3^/uL)	1.21	1.04	1.00	1.35	1.06	1.18	1.21	0.007	0.43	0.06–1.30
Basophils (10^3^/uL)	0.05	0.03	0.03	0.03	0.04	0.03	0.04	0.003	0.45	0.00–0.10
Platelets (10^3^/uL)	137	144	163	208	145	203	163	8.944	0.06	148–484
RDW (fL)	34.1	32.3	34.4	34.0	31.8	33.1	34.2	0.350	0.22	9.10–19.4
Blood urea nitrogen (mg%)	11.7	12.5	12.8	12.1	12.7	12.1	11.1	0.252	0.33	10.0–26.0
Creatinine (mg%)	1.10^bc^	1.21^cd^	1.16^bcd^	1.03^ab^	0.95^a^	1.25^d^	1.05^ab^	0.018	<0.001	0.50–1.30
ALT (IU/L)	44.1	30.4	33.9	25.1	30.7	31.3	34.6	2.784	0.68	6.00–70.0
Total protein (g%)	6.29	6.40	6.15	6.41	6.34	6.13	8.15	0.269	0.55	5.30–7.80
Albumin (g%)	3.00	2.82	3.00	2.65	2.68	2.91	3.09	0.046	0.06	2.30–3.20

1 MCV=Mean corpuscular volume, MCHC=Mean corpuscular hemoglobin concentration, MCH=Mean corpuscular hemoglobin, RDW=Red cell distribution width, MPV=Mean platelet volume, ALT=Alanine aminotransferase.

2 Different superscript letters in the same row represented the statistically significant differences (p<0.05).

3 Group 1=Control group (none of probiotic supplementation), Group 2=*plantarum* CM20-8 (TISTR 2676), Group 3=*Lactobacillus acidophilus* Im10 (TISTR 2734), Group 4=*Lactobacillus rhamnosus* L12-2 (TISTR 2716), Group 5=*Lactobacillus paracasei* KT-5 (TISTR 2688), Group 6=*Lactobacillus fermentum* CM14-8 (TISTR 2720) and Group 7=Mixed of probiotics.

4 Reference intervals were derived from the Veterinary Diagnostic Laboratory Kasetsart University Veterinary Teaching Hospital, Faculty of Veterinary Medicine, Kasetsart University, Bangkok, Thailand

### Fecal ammonia, pH, digestive enzymes, and digestibility

Fecal ammonia and pH did not differ among the groups (p > 0.05). Fecal digestive enzyme activities of amylase, protease, and cellulase also did not show any significant differences among the groups (p > 0.05). The results for the apparent digestibility of dry matter and nutrients were also not different among the groups. However, dogs supplemented with *L. plantarum* CM20-8 (TISTR 2676) appeared to present higher organic matter, crude protein, and ether extract digestibility than the other groups. Nevertheless, no statistically significant differences were found (p > 0.05). All of these data are presented in Table-3.

**Table-3:** Effects of probiotic addition in dog food on fecal ammonia, fecal pH, fecal digestive enzymes and apparent digestibility on dry matter and nutrients.

Parameters	Groups1							SEM^2^	p-value

	1	2	3	4	5	6	7		
Fecal ammonia (%)	0.18	0.17	0.18	0.16	0.18	0.18	0.18	0.005	0.78
Fecal pH	6.19	6.13	6.04	5.89	6.17	6.02	6.28	0.051	0.30
Fecal digestive enzyme activity									
Amylase (U)	0.77	0.93	0.95	0.78	1.00	0.81	0.86	0.107	0.37
Protease (U)	0.13	0.19	0.13	0.16	0.20	0.21	0.19	0.028	0.09
Cellulase (U×10^-2^)	2.27	2.26	2.62	2.62	2.29	2.70	2.06	0.002	0.19
Apparent Digestibility (%)									
Dry matter	75.4	80.7	73.9	72.6	73.2	74.9	72.8	0.911	0.22
Organic matter	77.8	82.6	76.6	75.2	75.9	77.3	75.4	0.838	0.23
Crude protein	71.8	77.4	69.2	68.4	68.5	70.2	69.4	1.070	0.28
Ether extract	88.2	92.5	89.6	88.0	89.2	89.2	88.2	0.509	0.22

^1^Group 1=Control group (none of probiotic supplementation), Group 2=*Lactobacillus plantarum* CM20-8 (TISTR 2676), Group 3=*Lactobacillus acidophilus* Im10 (TISTR 2734), Group 4=*Lactobacillus rhamnosus* L12-2 (TISTR 2716), Group 5=*Lactobacillus paracasei* KT-5 (TISTR 2688), Group 6=*Lactobacillus fermentum* CM14-8 (TISTR 2720) and Group 7=Mixed of probiotics

2 Standard error of mean

### Serum IgG and fecal IgA

Immunity in dogs was evaluated by assaying serum IgG and fecal IgA (Table-4). Serum IgG and IgA did not differ significantly among the groups (p > 0.05). However, serum IgG and fecal IgA in dogs supplemented with *L. paracasei* KT-5 (TISTR 2688) increased from days 0 to 14 of the experimental period when compared with the control.

**Table-4:** Effects of probiotic addition in dog food on serum IgG and fecal IgA on days 0 and 14.

Parameters	Groups^1^							SEM	p-value		
	
	1	2	3	4	5	6	7		Group	Time	Group*time
Serum IgG (mg/mL)											
Day 0	59.9	63.7	46.0	72.6	60.2	54.4	64.0	4.428	0.78	0.68	0.66
Day 14	56.8	66.1	55.8	57.0	80.9	60.8	59.1	3.523			
Fecal IgA (mg/mL)											
Day 0	193	219	193	286	405	383	347	40.21	0.40	0.43	0.99
Day14	266	248	175	324	536	391	367	44.58			

1 Group 1=Control group (none of probiotic supplementation), Group 2=*Lactobacillus plantarum* CM20-8 (TISTR 2676), Group 3=*Lactobacillus acidophilus* Im10 (TISTR 2734), Group 4=*Lactobacillus rhamnosus* L12-2 (TISTR 2716), Group 5=*Lactobacillus paracasei* KT-5 (TISTR 2688), Group 6=*Lactobacillus fermentum* CM14-8 (TISTR 2720) and Group 7=Mixed of probiotics, Ig=Immunoglobulin

## Discussion

Safety is a crucial characteristic of each probiotic strain applied in animal studies [58]. In a previous study, *Lactobacillus* strains were safe for a rat model. [59]. In this study, we did not find abnormal clinical signs until the end of the experiment. The hematology and serum biochemistry results of the *L. paracasei* KT-5 (TISTR 2688) and *L. fermentum* CM14-8 (TISTR 2720) groups revealed a difference for creatinine (p < 0.001), but their values were within the normal reference range. This confirmed the safety and non-pathogenicity of the new strain of *Lactobacillus* spp. in dogs at a dose of 10^9^ CFU per day for 28 days. The results are in close agreement with the previous research on the safety of the probiotic *Lactobacillus salivarius* CECT 5713 and other strains of *Lactobacillus* in mice [60, 61]. Furthermore, the use of *Lactobacillus fermentum* NCIMB 41636, *L. plantarum* NCIMB 41638, *L. rhamnosus* NCIMB 41640, and *Lactobacillus reuteri* was not associated with any dangerous clinical signs in dogs [62, 63].

In our study, no significant changes were observed in body weight, feed intake, body condition score, fecal score, and fecal moisture between the studied groups. This is in agreement with the previous studies concerning the effects of *L. acidophilus* D2/CSL (CECT 4529) on nutritional and health status (body weight, feed intake, and body condition score) in dogs [22, 64] and cats [65]. *Lactobacillus plantarum* (3.0 × 10^8^ CFU/mL) administered for 28 days did not change serum total cholesterol, WBC count, RBC count, or the blood lymphocyte percentage in healthy beagles [25]. Some authors have shown a positive effect of *Lactobacillus* strains in unhealthy dogs related to the prevention and treatment of acute gastroenteritis [66], inflammatory bowel diseases [36], and diarrhea [67]. A previous study reported an anti-obesity effect of *Lactobacillus gasseri* (LG2055) through the prevention of body weight gain, fat accumulation, and proinflammatory gene expression in the adipose tissue of obese mice [68]. Furthermore, the connection of obesity with serotonin hormone and the gut microflora in dogs was examined since this describes how obesity is related to neuron signaling in the brain [69]. The previous research supported the idea that intestinal microbiota may be associated with the control of fat accumulation in dogs [69, 70]. From the data provided in the literature, we propose that the addition of probiotics may preferentially maintain the equilibrium of the microbiota and control weight in dogs. However, further studies should investigate this in the intestinal microbiota.

*Lactobacillus plantarum* D2/CSL provided in healthy dogs for 35 days at a concentration of 5 × 10^9^ CFU/g improved fecal moisture, fecal score, and fecal hardness [22], and *L. acidophilus* strain DSM13241 (>10^9^ CFU/g) enhanced RBC count, hematocrit, and IgG for 4 weeks [23]. Although there were no differences among the groups in fecal scores and fecal moisture, fecal moisture on day 28 of *L. plantarum* CM20-8 (TISTR 2676) and *L. fermentum* CM14-8 (TISTR 2720) tended to be reduced (p = 0.08). Fecal ammonia and pH did not differ significantly among the groups. These data are in agreement with a report by Swanson *et al*. [13], who used *L. acidophilus* in dogs at a dose of 10^9^ CFU. Nevertheless, *L. acidophilus* (3.0 × 10^8^ CFU/mL; 10 mL/dog) administered for 28 days reduced fecal ammonia in healthy beagle dogs [25]. Kumar *et al*. [30] reported that *L. johnsonii* CPN23 at a dose of 2–3 × 10^8^ CFU/day decreased ammonia production in adult female Labrador dogs for 9 weeks. These results differed from ours because the dose and duration used in the previous *Lactobacillus* studies were greater than those used in the present study. Fecal digestive enzyme activity and digestibility were not significantly affected by LAB. However, *L. plantarum* CM20-8 (TISTR 2676) appeared to present higher numerical values for organic, crude protein, and ether extract digestibility. In dogs, *L. johnsonii* CPN23 at a dose of 2–3 × 10^8^ CFU/day did not improve digestibility apart from that of fiber [30]. The results were similar to previous studies, in which *L. plantarum* (1.2 × 10^9^ CFU) in chickens and *L. plantarum* BG0001 in weaning pigs did not promote digestibility [71, 72]. There was a tendency for a significant upward trend in proteinase activity (p = 0.09) in the *L. plantarum* CM20-8 (TISTR 2676) group, supporting the higher digestibility of this taxon. Immunoglobulin A contains numerous classes of antibodies involved in mucosal secretion. It confers many benefits, such as infection prevention and protection against allergens and is important for evaluating mucosal immune status [73]. Another marker used as an indicator of immune status is serum IgG. It reduces bacterial translocation, intestinal damage, and systemic infection by binding to bacteria in the intestinal lumen [74]. Immune function in dogs was assessed through serum IgG and fecal IgA, which were not found to differ among the groups. Although other studies increased fecal IgA [75], IFN-α, and serum IgG content [33], in a study with *Lactobacillus kefiri* [76] and *L. acidophilus*, fecal IgA level was not changed [77]. Serum IgG and fecal IgA in dogs supplemented with *L. paracasei* KT-5 (TISTR 2688) increased from days 0 to 14 of the experimental period when compared with those in control. In contrast, the mixture of probiotics had no significant effect over time.

## Conclusion

Although supplementation with new *Lactobacillus* strains *L. plantarum* CM20-8 (TISTR 2676); *L. acidophilus* Im10 (TISTR 2734); *L. rhamnosus* L12-2 (TISTR 2716); *L. paracasei* KT-5 (TISTR 2688); *L. fermentum* CM14-8 (TISTR 2720); and mixed probiotics] did not change nutritional status, enzyme activities, and immunity in dogs, they were found to be safe and non-pathogenic in dogs. No changes in body weight, feed intake, or body condition score were found. However, further studies should investigate the intestinal microbiota and the development of associated clinical treatments.

## Authors’ Contributions

KP: Conceptualization, prepared materials, conducted the study, analysis, interpretation, drafted and revised the manuscript. SA, PiC, PPa, NA, PT, PC, KN and PPh: Conceptualization, prepared materials, conducted the study, analysis, interpretation and revised the manuscript, JS and AK: Conceptualization, project administration, conducted the study, data interpretation and revised the manuscript. All authors have read, reviewed, and approved the final manuscript.
